# Feeling One Thing and Doing Another: How Expressions of Guilt and Shame Influence Hypocrisy Judgment

**DOI:** 10.3390/bs12120504

**Published:** 2022-12-09

**Authors:** Hyeman Choi

**Affiliations:** Department of Psychology, Gachon University, Seongnam 13120, Republic of Korea; choih@gachon.ac.kr; Tel.: +82-31-750-2663

**Keywords:** shame, guilt, future change, emotion expression, hypocrisy, redemption, moral judgment, self-conscious emotion, emotion communication

## Abstract

The present study investigated how people, as uninvolved social observers (i.e., those not affected by the emotion expresser’s behavior), judge hypocrisy in a target who publicly expresses their self-conscious emotions (i.e., shame and guilt) after making an immoral decision, then repeats the same immoral behavior again. Results across the two studies conducted showed that participants viewed the target as more hypocritical when the target expressed guilt (vs. shame) for their past misdeed and then committed the same act again. The present study suggests that social perceivers tend to infer expressions of guilt (and of shame to a lesser degree) as signaling future changes, which is reflected in judgments of hypocrisy. The study further discusses implications for the social functions of emotional expression and communication.

## 1. Introduction

Researchers of emotion have been successful in distinguishing and determining the nuances of shame and guilt, particularly in terms of how the two self-conscious emotions are experienced differently (see [1] for a review). However, little is known about what people, especially as uninvolved social observers (i.e., those not affected by the emotion expresser’s behavior), expect from targets who have communicated shame and guilt. In particular, despite rigorous findings that guilt is more conducive to changing one’s behavior in the future [1], it is still unknown whether people expect greater future behavioral changes in others when they express guilt than when they express shame. Therefore, the present study tested whether people hold a naïve theory about others’ future changes in behavior, that is, that a person who expresses guilt will change their behavior to a significant degree. It was expected that people’s understanding of the link between others’ self-conscious emotions and future change would be reflected in their judgments of hypocrisy regarding others.

### 1.1. Implications of Guilt and Shame for Future Behaviors

Although both guilt and shame are painful emotions that stem from past misdeeds that fall short of one’s own or others’ standards [2,3,4], the extant research suggests that guilt has greater potential for affecting future change because guilt and shame arise from disparate appraisals and have different motivational consequences [1]. Focusing on such appraisals, guilt results from the appraisal of one’s own wrong behavior, especially in the context of an event involving other people [5,6,7], whereas shame results from the evaluation of the defects in the self [8]. Consistent with this distinction, Tracy and Robin [9] showed that people tend to experience guilt for unstable, controllable, and specific aspects of the self that are amendable (e.g., lack of effort), whereas they experience shame for stable, uncontrollable, and global aspects of the self that are difficult to change (e.g., lack of ability). These cognitive differences imply that guilt has a greater potential for affecting future changes in behavior than shame. It is much easier to change a specific aspect or idiosyncrasy of one’s behavior than to change one’s entire character or personality. Thus, given that guilt arises when individuals focus on specific and concrete behavior, whereas shame is experienced when they focus on abstract self-aspects [8], it seems clear that experiencing guilt has a greater potential for affecting future change in behavior [1]. The difference in motivational consequences between guilt and shame also contributes to differences in the implications for future behavioral change. Guilty feelings motivate individuals to approach and empathize with the target and correct situations involving others [5,10]. In contrast, feeling shame motivates individuals to avoid and withdraw from the shame-eliciting situation [1,11]. Being motivated to approach the guilt-eliciting situation, a guilty person creates an opportunity to correct past misdeeds or recover from broken relationships. Therefore, the feeling of guilt is said to be a functional aspect of society, useful in regulating its cooperative system [12]. A person who is ashamed, however, may find it hard to have any opportunity to correct the situation because they are motivated to escape the situation. Thus, for both cognitive and motivational reasons, it seems reasonable to believe that guilt has a greater potential for affecting future behavior change. Compelling evidence comes from the study of young prisoners whose self-reported guilty experiences during their early days in prison predicted lower recidivism, whereas shameful experiences predicted higher recidivism [13,14].

Therefore, converging evidence shows that when individuals experience guilt (vs. shame), they are more likely to change their behavior. Notably, people tend not only to experience and express their own self-conscious emotions but also to observe others’ experiences of emotions and make inferences based on them.

### 1.2. Seeing Others’ Self-Conscious Emotions

Understanding how people perceive and interpret others’ feelings of shame and guilt is important, because each emotion has a different social function [12]. Emotions communicate a rich form of information, such as a person’s personality (e.g., [15]), how a person responds to a given stimulus [16], and what an effective interaction strategy for a target person would look like (e.g., [17,18,19]). What social meanings do shame and guilt communicate in the context of affecting change in the future? Given that people, as social observers, have inherently limited access to the inner states of others, they have to extrapolate their own feelings to understand what others would feel and how they would react in a given situation [2,20]. Therefore, based on their own experience, people would expect guilt-experiencing individuals to be motivated to change or improve their behavior [21]; in contrast, people may think that expressions of shame do not necessarily mean that the target would change their behavior. In support of this argument, Giner-Sorolla and Espinosa [22] found that people think of guilt as a more appropriate emotion to feel when others express anger (vs. disgust) in front of them, whereas people consider shame a more appropriate emotion to feel when others express disgust (vs. anger), indicating that guilt is more apt at communicating one’s intention and effort (i.e., changing one’s behavior) to repair an unfair situation with the aim of soothing the angered party. In a related vein, exhibiting remorse, an emotion that reflects perceived responsibility for past wrongs, is effective in alleviating the severity of punishment in the courtroom, helping people believe that a truly remorseful individual would not commit the same criminal act again [23,24]. For the same reason, offering an apology and showing remorse also helps increase the likelihood of forgiveness by others [25,26,27]. Interestingly, guilt (as opposed to shame) is conceptually closer to remorse [23,28] and wanting to apologize is the motivational state of guilt [1,29], suggesting that guilt would probably signal similar social meanings (i.e., an intention to make reparations) to what remorse and apologies convey. This means that guilt is more likely to engender the effects of remorse and apology.

Indeed, Stearns and Parrott [30] investigated whether displays of guilt and shame influence the perception of people by uninvolved observers. Results showed that participants in both situations viewed the target person as more moral and likeable compared to situations of no emotional response (i.e., “I don’t feel bad about it”). Importantly, the measure of moral character, which did not differ between conditions of guilt and shame, contained an item asking whether participants believed that the target would behave in the same way in a similar situation in the future. Stearns and Parrott [30] concluded that people seemed not to fully differentiate the social meanings of guilt and shame based on the mere expression of guilt and shame, namely the usage of the terms, ‘guilt’ and ‘shame’ (study 1), or the characteristic appraisals of each emotion (study 2). In line with this, research has shown that an apology accompanied by feelings of guilt and shame was equally effective in increasing the likelihood of forgiveness [31]. Thus, in the extant literature, it seems unclear whether people infer greater future change from expressions of guilt than from expressions of shame. The present study argues that the undifferentiated effect of shame and guilt in previous studies may stem from the nature of the dependent variables (i.e., moral judgment and forgiveness), which are less sensitive to measuring the implications for future change. In other words, moral judgment and forgiveness involve more complex mental processes than simple expectations of future change, inferred from the emotions of others. The difference in social meanings with respect to the potential for future change elicited by guilt on the one hand and shame on the other would be captured best in a research paradigm that provides a specific context for the future, wherein the implications for future behavior can be said to be meaningful. Therefore, this study employed a future-relevant paradigm to differentiate between guilt and shame regarding future change from the perceiver’s perspective.

### 1.3. The Present Study: Feeling One Thing and Doing Another

The perception of hypocrisy regarding others provides a good context for testing the difference in the implications of shame and guilt for future behavior because the judgment of hypocrisy involves the issue of inconsistency [32,33]. Just as people see themselves as hypocrites when confronted with inconsistencies among their behaviors [34], judging others as hypocrites involves perceived behavioral discrepancies within the target person (cf. [35]). For example, Barden et al. [32] showed that people think of a target person as more hypocritical when the target person makes a public statement proclaiming one thing (e.g., “virtue of observing laws”) and then does another (adopting an expedient) than when the person does one thing and then says another. This order effect on hypocrisy judgment was mediated by participants’ impression that the target person had reformed. In other words, people expect a redemptive story (i.e., moral improvement) from the target when the target publicly states personal standards after committing immoral behavior. On the contrary, the case of saying-one-thing-and-doing-another creates a state of expectation violation, because people assume that what others say reflects their values and standards and, therefore, expect others to behave in a manner consistent with what they say.

Likewise, people think that a target who expresses self-conscious emotions in public for what he or she has done in the past (i.e., doing one thing and feeling another) would not commit the same act again [30]. Consequently, people do not view the target as a hypocrite. However, such expectations for future change will be violated when the target re-engages in past wrong behavior after expressing those emotions (i.e., feeling one thing and doing another), leading the perceivers to view the target as hypocritical. Presumably, hypocrisy perception is proportional to the degree of perceived personal inconsistency (cf. [36]). Thus, given that guilt, rather than shame, signals greater potential for future change, the hypothesis is that a protagonist who repeats immoral behavior after expressing guilt rather than shame in public (i.e., recursion condition) would be viewed as more hypocritical. On the contrary, there would be no such difference in the hypocrisy judgment for the redemption condition in a situation in which no further information about behavior is given, as no expectation would be violated. In addition, one item that directly measured expectations of future change was included. Although Stearns and Parrott [30] did not find a difference, considering the preceding analysis, it was expected that participants in the guilt-redemption (vs. shame-redemption) condition would report greater expectations of future change.

## 2. Study 1

### 2.1. Method

#### 2.1.1. Participants and Design

A total of 242 participants from an online survey website (Amazon Mechanical Turk) participated in Study 1 (*M*_age_ = 34.79, *SD* = 11.88; 61.2% female; 71.5% Caucasian, 13.6% African American, 7.4% Hispanic/Latino, 5.4% Asian/Asian American, and 2.1% other). Participants were randomly assigned to the conditions of a 2 (event meaning: redemption vs. recursion) × 3 (emotion type: guilt vs. shame vs. control) between-participants design (*n* = 39–41 per cell).

#### 2.1.2. Procedure and Materials

Participants read a short scenario (see Appendix A) that described a chairman who makes a final decision on a proposed new project for their company that, while increasing the company’s profits, would harm the environment. This scenario was modified from the version employed by Knobe [37]. The type of emotion the chairperson expressed was manipulated either as guilt (“*I feel guilty about my decision*”), shame (“*I feel ashamed about my decision*”), or control (i.e., no emotional expression). The event meaning (redemption vs. recursion) was also manipulated such that participants under the recursion condition read of a scenario wherein the chairman made a similar decision two weeks after the previous committee meeting, where he expressed guilt or shame in front of other committee members (or no information, for the control condition). Participants under the redemption condition read the same scenario, but the story did not include any information on the second decision. Then, using a 7-point scale (1 = *not at all*, 7 = *absolutely*), participants rated the guilt and shame intensity (“*How guilty/ashamed do you believe the chairman was feeling for making the decision to start the new program?*”) and degree of the chairman’s motivation to change their attitude toward the company’s role in protecting the future ecosystem. Finally, participants rated how hypocritical they found the chairman (1 = *not at all*, 7 = *absolutely*).

### 2.2. Results

#### 2.2.1. Shame and Guilt Intensity

A 2 (event meaning: redemption vs. recursion) × 3 (emotion type: guilt vs. shame vs. control) analysis of variance (ANOVA) performed on the metric of guilt intensity revealed significant main effects of event meaning (*F*(1, 236) = 94.92, *p* < 0.001, η_p_^2^ = 0.287) and emotion type (*F*(2, 236) = 8.03, *p* < 0.001, η_p_^2^ = 0.064), as well as an interaction effect (*F*(2, 236) = 6.16, *p* = 0.002, η_p_^2^ = 0.05). The simple effect of emotion within the redemption condition showed that participants under the guilt (*M* = 5.03, *SD* = 1.73) and shame (*M* = 4.76, *SD* = 1.73) conditions viewed that the chairman experienced greater guilt than did those under the control condition (*M* = 3.22, *SD* = 2.02) (*F*(2, 236) = 11.72, *p* < 0.001, η_p_^2^ = 0.05). For the recursion condition, the simple effect of emotion type was marginally significant (*F*(2, 236) = 2.47, *p* = 0.087, η_p_^2^ = 0.05), converging low on the scale: guilt (*M* = 1.75, *SD* = 1.35), shame (*M* = 2.56, *SD* = 1.94), and control (*M* = 1.93, *SD* = 1.93). Second, for rating the intensity of shame, the same analysis revealed significant main effects of event meaning (*F*(1, 236) = 104.59, *p* < 0.001, η_p_^2^ = 0.307) and emotion type (*F*(2, 236) = 15.22, *p* < 0.001, η_p_^2^ = 0.114), but no interaction effect (*F*(2, 236) = 1.69, *p* = 0.188, η_p_^2^ = 0.014), such that participants viewed the protagonist as experiencing more intense shame under the redemption condition (*M* = 4.17, *SD* = 1.96) than under the recursion condition (*M* = 1.96, *SD* = 1.59). Participants under both guilt (*M* = 3.61, *SD* = 2.23) and shame conditions (*M* = 3.39, *SD* = 2.09) rated higher on shame intensity items than did those under the no-information condition (*M* = 2.24, *SD* = 1.07).

#### 2.2.2. Change Expectation

The same 2 × 3 ANOVA on the participant’s ratings of the change expectation yielded a significant main effect of event meaning, such that participants under the redemption condition (*M* = 4.60, *SD* = 1.88) believed that the chairman would change his attitude toward the role of the company in protecting the environment to a greater degree than those in the recursion condition (*M* = 2.36, *SD* = 1.84) (*F*(1, 236) = 88.25, *p* < 0.001, η_p_^2^ = 0.272). Neither the main effect of emotion (*F* < 1) nor the event meaning × emotion type interaction was significant (*F*(2, 236) = 1.92, *p* = 0.149, η_p_^2^ = 0.016).

#### 2.2.3. Hypocrisy Judgment

**Figure 1:**
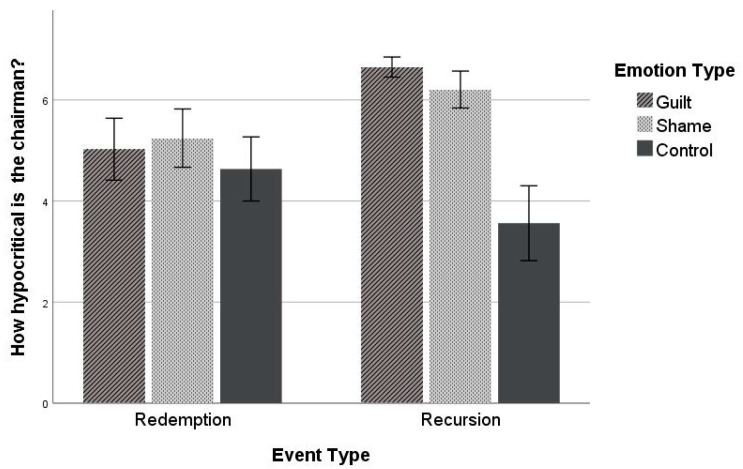
Hypocrisy ratings (Study 1). Error bars represent standard errors.

The single-item hypocrisy judgment was subjected to a 2 × 3 ANOVA. The results showed that there were significant main effects of event meaning (*F*(1, 236) = 5.03, *p* = 0.026, η_p_^2^ = 0.021) and emotion type (*F*(2, 236) = 25.21, *p* < 0.001, η_p_^2^ = 0.176). However, the main effects were qualified by a significant event meaning × emotion type interaction effect (*F*(2, 236) = 13.13, *p* < 0.001, η_p_^2^ = 0.10). Supporting the hypothesis, a planned comparison of the relevant means showed that within the recursion condition, participants under the guilt condition (*M* = 6.65, *SD* = 0.62) judged the chairman to be more hypocritical than those under the shame condition (*M* = 6.21, *SD* = 1.13) (*t* = 5.32, *p* < 0.001, *d* = 0.53, 95% CI [1.13, 2.47]). Participants under both the guilt and the shame conditions rated the target as more hypocritical than those under the control condition (*M* = 3.56, *SD* = 2.35) (*p*s < 0.001). For the redemption conditions, hypocrisy ratings did not differ across conditions (*M*_redemption_ = 4.97, *SD* = 1.92) (*F*(2, 236) = 1.28, *p* = 0.279 (see Figure 1)).

### 2.3. Discussion

Study 1 tested the link between perceptions of self-conscious emotion expression and how people judge someone as hypocritical. Supporting the hypothesis, the results suggest that people judge a target as more hypocritical when the individual publicly expresses guilt (vs. shame) and then engages in the same behavior again. Importantly, the hypocrisy rating did not differ under the guilt, shame, and control conditions when there was no information about the following similar decision by the chairperson; therefore, participants’ expectations had not been violated (i.e., the redemption condition). On the contrary, the judgement of hypocrisy under the guilt and the shame conditions departed from the control condition to a different degree when participants’ expectations had been violated, as the guilt- or shame-expressing person commits the same kind of behavior again (i.e., recursion condition). These results provide clear evidence of people expecting future change of a different degree depending on the emotion (i.e., shame or guilt) a target expresses.

As for the null effect of the manipulations on the expectation measure, it may be that, as in Stearns and Parrott [30], the present findings reflect the true state of reality, wherein people cannot or do not differentiate guilt from shame with regards to face value and in the context of future behavior. It could be that, when participants in this study read the terms “guilt” or “shame”, they automatically construed the term as the guilt–shame pair, thinking that guilty and shameful feelings go together, which, in turn, weakens the unique effect of each emotion [1,30,38]. Another possibility is that the wording of the change expectation item (i.e., attitude toward business ethics) did not specify the behavioral change (i.e., not approving a similar proposal in the future that runs the risk of harming the environment), which could have diluted the effect of the manipulation. Study 2 attempts to address these issues.

## 3. Study 2

To account for the possible nullifying effect of using the terms guilt and shame, Study 2 used the characteristic appraisals of guilt and shame instead (see Appendix A) [1,30]. Moreover, guilt and shame intensity items were presented at the end of the questionnaire packet so that participants were not exposed to terms such as guilt or shame before they rated the expectation and hypocrisy judgment items. With regard to the change-expectation measure, Study 2 forced participants to choose an option that best described their expectations of the chairperson’s behavioral change.

### 3.1. Method

#### 3.1.1. Participants and Design

A total of 250 participants (*M*_age_ = 35.82, *SD* = 12.56; 41.7% male, 58.3% female; 73.3% Caucasian, 8.5% African American, 8.1% Asian/Asian American, 7.3% Hispanic/Latino, 2.8% other) from an online survey website (Amazon Mechanical Turk) were randomly assigned to conditions of a 2 (event meaning: redemption vs. recursion) × 3 (emotion type: guilt vs. shame vs. control) between-participants design (*n* = 40–44 per cell).

#### 3.1.2. Procedure and Materials

The participants read a scenario identical to the one used in Study 1, except for the description of the emotional expression made by the chairman. Participants under the guilt condition read:

*“I feel bad about making the decision to start the new program and causing her suffering. I would like to contact her right now and apologize for what I have done. I just want to tell her that I am sorry for what I have done, and that I would like to help make things right.”*

Participants under shame condition read:

*“I feel bad about making the decision to start the new program and causing her suffering. I think I am a terrible person for what I have done, and I feel like I just want to hide right now.”*

Subsequently, participants were asked to select one of two options (i.e., “*I think the chairman would approve/reject similar proposals in the future*.”) that reflected their expectations of the chairperson’s behavior in the future. Then, participants rated the hypocrisy item. Finally, participants rated how intensely they expected the chairperson to experience feelings of guilt and shame.

### 3.2. Results

#### 3.2.1. Change Expectation

Within the redemption condition, 61.9% (*n* = 26) of participants under the guilt condition and 80.5% (*n* = 33) under the shame condition believed that the chairman would not make the same decision again in the future, whereas 27.5% (*n* = 11) of participants under the control condition believed the same claim, *χ*^2^(2) = 23.83, *p* < 0.001. However, within the recursion condition, Fisher’s exact test showed that only a few participants believed that the chairman would change his mind in the future, regardless of the emotion condition type they were under: 10% (*n* = 4) for guilt, 7% (*n* = 3) for shame, 2.3% (*n* = 1) for no information, *χ*^2^(2) = 2.17, *p* = 0.312.

#### 3.2.2. Hypocrisy Judgment

**Figure 2:**
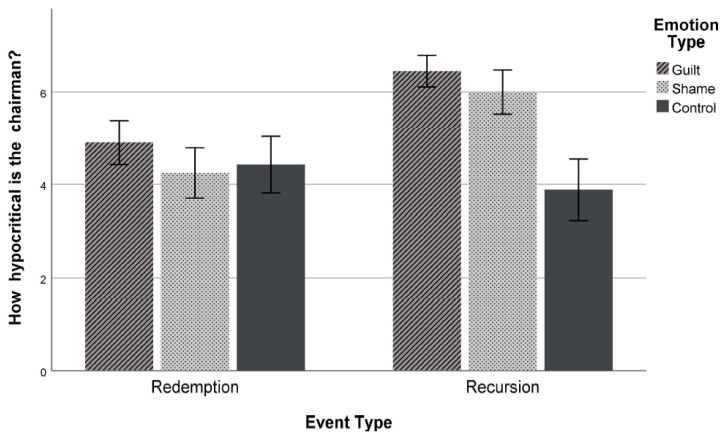
Hypocrisy ratings (Study 2). Error bars represent standard errors.

A 2 × 3 ANOVA on hypocrisy judgment revealed significant main effects of event meaning (*F*(1, 244) = 18.42, *p* < 0.001, η_p_^2^ = 0.07) and emotion type (*F*(2, 244) = 17.14, *p* < 0.001, η_p_^2^ = 0.123), as well as a significant event meaning × emotion type interaction effect (*F*(2, 244) = 11.70, *p* < 0.001, η_p_^2^ = 0.088). Supporting the hypothesis and replicating the findings of Study 1, comparisons of relevant means showed that participants under the recursion condition viewed the chairman as more hypocritical when the chairman expressed guilt (*M* = 6.45, *SD* = 1.06) than shame (*M* = 6.00, *SD* = 1.54) (*t* = 4.57, *p* < 0.001, *d* = 0.25, 95% CI [84, 2.12]). Participants under both the guilt and the shame conditions rated the target as more hypocritical when compared with the control condition (*M* = 3.89, *SD* = 2.16) (*p*s < 0.001). For the redemption condition, hypocrisy ratings did not differ across conditions (*M*_redemption_ = 4.53, *SD* = 1.73) (*F*(2, 244) = 1.61, *p* = 0.201 (see Figure 2)).

### 3.3. Discussion

Replicating Study 1, Study 2 demonstrates that people think of a target as more hypocritical when said target expresses guilt rather than shame, followed by an inconsistent act. Identical to the findings of Study 1, the hypocrisy judgment diverged only when participants’ expectations were violated (i.e., under the recursion condition). This reflects people’s prevalent impression that a guilt-ridden person would try to change his or her behavior and make a more moral decision in the future, as opposed to a person filled with shame.

## 4. General Discussion

The present study sought to establish a relationship between others’ expressions of self-conscious emotions and the observer’s theory of mind. By manipulating the type of expressed self-conscious emotions and the meaning of the event, this study demonstrated that the expression of guilt (vs. shame) followed by the same immoral behavior leads to a greater level of hypocrisy perception by uninvolved observers, indicating that people expect a person to show greater future behavior change from their expressions of guilt than of shame. It should be noted that in Study 2, consistent with the findings of Study 1 and that of Stearns and Parrott [30], the difference in the direct change-expectation ratings between shame and guilt conditions was not significant. It appears, then, that additional contextual information, for example, personal-consistency information, as in the present study, is critical in distinguishing subtle differences in the social meaning of guilt and that of shame.

The present study contributes to the extant literature on emotions by exploring the observer’s perspective of the expression of guilt and shame by other people, which is a relatively under-studied subtopic in this field. Although some researchers have speculated (e.g., [39]) and indeed tested (e.g., [30,31]) the idea that guilt signals greater potential for future change to social observers when opposed to shame, no empirical evidence had been reported so far. Thus, the present study is the first to demonstrate the systematic difference in people’s naïve theories of shame and guilt and the contingency of future change upon the former. Indeed, differentiating often similarly experienced emotions would be even more challenging from the observer’s perspective (e.g., [7]) because shame and guilt are similar in the eyes of observers who tend to construe others at a more abstract level than themselves [40]. The present study suggests that placing guilt and shame in a future context could be an effective way of navigating the distinctive nuances in the social meanings of guilt and shame. By manipulating shame and guilt in a future-specific context, this study distinguished the implications of guilt and shame for future changes in the eyes of observers. Furthermore, the present study highlights the moral standing of self-conscious emotions from the perspective of social observers. In the literature on emotion, guilt is considered a typical moral emotion because it promotes moral motivation [41,42,43] while shame is considered to have a less-clear identity as a moral emotion. In line with the social-functional perspective [12], this study suggests that the expression of guilt (vs. shame) is more valued by others, especially when the same event is expected to reoccur, because guilt signals a promise for future change, a reassurance missing from the symbolic perception of shame. Consequently, some individuals may be tempted to exaggerate their guilty feelings, the “correct” emotion [22], in the hope they could then receive greater forgiveness from the affected victim or uninvolved social others. However, this study suggests that expressing guilt may risk one being perceived as a hypocrite when one commits the same act again. In such cases, exhibiting remorse would lead to more negative evaluations of the person because people would see the person as insincere (e.g., [44,45]). Considering that hypocrisy judgment is closely related to feelings of trust, the present findings will help us understand how expressions of shame and guilt, along with other situational factors, differentially influence the perceived authenticity of remorse, especially in legal settings (e.g., [46]).

### Limitations and Future Directions

The present study has several limitations. First, sharing the same problem with other studies that used a scenario method (e.g., [47]), a brief description of the moral situation in the present study might be less detailed than necessary, which might have undermined the effect of the independent variables. Social observers received only a limited amount of information. Future studies could employ more evocative stimuli, such as video clips or images of emotion expressions [48], with a detailed description of the moral situation. Second, the moral situations used in the present study were mild moral transgressions. A meta-analysis by Proeve and Tudor [23] showed that the effect of remorse felt by an offender on alleviating the level of punishment tends to be stronger for less serious offences (e.g., cheating) than for more serious offences (e.g., manslaughter). Thus, the current findings may only hold true for mild transgressions.

## 5. Conclusions

Studying the perceptions of others’ shame and guilt is important because it reveals the social functions and underlying motivations for experiencing and communicating self-conscious emotions. The present study therefore fills an important gap in the extant literature on emotions. I hope that this line of research will invite more researchers to examine various aspects of communicating self-conscious emotions.

## Data Availability

The data presented in this study are available upon request from the corresponding author.

## Appendix A

### Appendix A.1. Scenario in Study 1

(for all conditions) The vice-president of a company went to the chairman of the board and said, “We are thinking about starting a new program. It will help us increase profits, but it will also harm the environment, specifically the soil conditions.” The chairman of the board answered, “I don’t care at all about harming the environment. I just want to make as much profit as I can. Let’s start the new program.” The company started the new program and sure enough, the environment was harmed. A few months later, the chairman heard the news about people becoming violently ill after eating crops that were grown on a contaminated farm.

(for guilt/ashamed conditions only) During the next board meeting, the chairman reflected on his decision for a while and said, “I feel guilty [ashamed] about making the decision to start the new program.”

(for recursion conditions only) Two weeks later, the vice-president of the company went to the chairman of the board again and said, “We have another new and promising program that will help us increase profits. But it may also harm the environment as we have seen in the past.” The chairman of the board answered, “I don’t care at all about harming the environment. I just want to make as much profit as I can. Let’s start the new program.”

The company started the new program.

### Appendix A.2. Scenario in Study 2

The vice-president of a company went to the chairman of the board and said, “We are thinking about starting a new manufacturing program. It will help us increase profits, but it will also harm the environment, specifically the soil conditions.” The chairman of the board answered, “I don’t care at all about harming the environment. I just want to make as much profit as I can. Let’s start the new program.” The company started the new program and sure enough, the environment was harmed. A few months later, the chairman was told about a woman named Chris who had fallen violently ill after eating crops that were grown on a contaminated farm. Chris, a 43-year-old woman who lived near the site where the company started the program, had recently been hospitalized for severe health problems that stemmed from abnormal heavy metal levels that had accumulated in her body.

(for guilt condition only) The next board meeting began with a discussion of Chris’ case. The chairman reflected on his decision for a while and then said, “I feel bad about making the decision to start the new program and causing her suffering. I’d like to contact her right now and apologize for what I’ve done. I just want to tell her that I’m sorry for what I’ve done, and that I’d like to help make things right.”

(for ashamed condition only) The next board meeting began with a discussion of Chris’ case. The chairman reflected on his decision for a while and then said, “I feel bad about making the decision to start the new program and causing her suffering. I think I’m a terrible person for what I’ve done, and I feel like I just want to hide right now.”

Two weeks later, the vice-president of the company went to the chairman of the board again and said, “We have another new and promising program that will help us increase profits. But it may also harm the environment as we have seen in the past.” The chairman of the board answered, “I don’t care at all about harming the environment. I just want to make as much profit as I can. Let’s start the new program.”

The company started the new program.
